# Genetic and Environmental Factors Associated to Glutenin Polymer Characteristics of Wheat

**DOI:** 10.3390/foods9050683

**Published:** 2020-05-25

**Authors:** Gérard Branlard, Annie Faye, Larbi Rhazi, Ayesha Tahir, Véronique Lesage, Thierry Aussenac

**Affiliations:** 1 The French National Research Institute for Agriculture, Food and the Environment (INRAE), UCA UMR1095 GDEC, 5 Chemin de Beaulieu, 63100 Clermont-Ferrand, France; gerard.branlard@gmail.com (G.B.); annie.faye@inrae.fr (A.F.); veronique.lesage@inrae.fr (V.L.); 2 Institut Polytechnique UniLaSalle, Université d’Artois, EA 7519, 19 rue Pierre Waguet, BP 30313, 60026 Beauvais, France; larbi.rhazi@unilasalle.fr; 3 Department of Biosciences, COMSATS University Islamabad, Park Road, Tarlai Kalan, 45550 Islamabad, Pakistan; ayesha.tahir2007@gmail.com

**Keywords:** wheat, glutenin polymers, environmental effects on polymer characteristics, NCGS

## Abstract

The polymers of wheat glutenins are studied here using asymmetric flow field flow fractionation (A4F). Molecular mass (Mw), gyration radius (Rw), and the polydispersity index (PI) of polymers were measured over a four-year, multi-local wheat trial in France. The experiment, involving 11 locations and 192 cultivars, offered the opportunity to approach the genetic and environmental factors associated with the phenotypic values of the polymer characteristics. These characteristics, which were all highly influenced by environmental factors, exhibited low broad-sense heritability coefficients and were not influenced by grain protein content and grain hardness. The 31 alleles encoding the glutenin subunits explained only 17.1, 25.4, and 16.8% of the phenotypic values of Mw, Rw, and PI, respectively. The climatic data revealed that a 3.5 °C increase between locations of the daily average temperature, during the last month of the grain development, caused an increase of more than 189%, 242%, and 434% of the Mw, Rw, and PI, respectively. These findings have to be considered in regard to possible consequences of global warming and health concerns assigned to gluten. It is suggested that the molecular characteristics of glutenins be measured today, especially for research addressing non-celiac gluten sensitivity (NCGS).

## 1. Introduction

The end-use quality of bread wheat (*Triticum aestivum* L.) and durum wheat (*Triticum durum* L.) are known to be largely influenced by the grain storage proteins (GSPs): gliadins and glutenins. Dough properties are influenced by the quantity of gliadins, which are monomeric and confer extensibility, whereas glutenins are polymeric and associated with dough strength and elasticity [1]. Since the seventies, many studies have focused on analysis of the genetic diversity of gliadins and glutenins. For bread wheat, these proteins are inherited in families of multi-genes clustered on 12 major loci on chromosomes of group 1 and 6 of the genomes A, B, and D [2]. Genetic and molecular analyses of GSPs, carried out in many countries, have clarified their influences on technological properties [3,4]. Some recent updates on wheat quality, particularly on the genetic, molecular, and technological attributes of these GSPs, which are essential components of the gluten, are reported in the international collaborative book [5]. Though the elementary bricks of the gluten are known, many factors involved in the quantitative expression of more than 100 of the genes and in the resulting polymers still remain to be investigated.

During grain development, the synthesis of GSPs is regulated by numerous transcription factors and by the availability of nitrogen and sulfur [6]. GSPs are synthesized in the endoplasmic reticulum and partly via the Golgi apparatus [7]. The proteins are accumulated in protein bodies, comprising a process of polymerization via interchain disulfide bonds (SS) of the high and low molecular weight glutenin subunits (HMW-GS and LMW-GS) and some α-, β-, and γ-gliadins, with an odd number of cysteines [8]. The polymers, mainly composed of glutenin polymeric proteins (GPP), also contain the monomeric gliadins aggregated by hydrogen bonding and hydrophobic interactions [2]. During grain development, the formed polymers exhibit an exponential phase of accumulation of their SDS-phosphate-insoluble fractions, which is revealed around 30 days after anthesis (daa) at the end of the water plateau, corresponding to the initiation of grain dehydration [9,10]. The compressed protein bodies release their material during dehydration/maturation of the grain, leaving the GSPs to surround the starch granules in the form of a fairly dense protein matrix. The kernel tissues are progressively engaged in programmed apoptosis [11].

Rather than using the electrophoresis techniques initially practiced to uncover the diversity of GSPs, high-performance liquid chromatography (HPLC) using size exclusion (SE) for molecular profiling has rapidly become the preferred tool for gluten sub-fraction analysis and quality characterization [12,13]. The determination of the percentage of unextractable polymeric proteins (%UPP), obtained after a two-step extraction using SDS followed by sonication, through SE-HPLC [14] was implemented, particularly in Sweden [15,16], but has also been used by many teams around world to approach some aspects of wheat quality and gluten strength or to evaluate influence of environmental factors on %UPP [17,18]. The %UPP provides information on polymer abundance, and this is sometimes correlated to grain protein concentration (PC) but does not describe the molecular characteristics of the polymers. The second technique for analyzing these polymers requires molecular sieving in a double cross-flow gradient followed by multi-angle detection of the deviation caused on a laser beam (A4F-MALLS, Asymmetric Flow Field-Flow Fractionation Multi-Angle Laser Light Scattering) [19]. This technique offers the possibility to measure several parameters (like molecular mass (Mw) and hydrodynamic radius) on each polymer or to have a distribution of these molecular characteristics on the analyzed sample and to calculate an index of polydispersity of Mw. All these measurements are not offered by SE-HPLC. The maximum size sieving permitted by chromatography columns is around 1 × 10^6^ Da, whereas polymer Mw higher than 1 × 10^7^ g/mol have been reported for GPPs [19,20]. The A4F-MALLS of GPP was used in several approaches, such as studying the quality stability of hybrid wheats over different environments [21], the influence of ozone treatment on wheat proteins and quality [22], or the effect of chitosan treatment on gluten proteins [23]. Using A4F-MALLS the influence of the genetic and/or environmental factors have initially reported [20] by studying in a limited set of cultivars grown on two years multilocal trials in France. These preliminary results were reported in workshop and conference proceedings. It was shown that the sum of the average daily air temperature in trial locations, cumulated over the two months of the grain-filling period (June, July), resulted in increased molecular mass of the glutenin polymers. The polymer mass increasing being higher for soft wheat as for hard wheat [24]. This also affected the technological quality in increasing the dough tenacity but decreasing dough extensibility and bread loaf volume [25]. This preliminary study, which involved only 2 °C of difference in daily average temperature between locations, are here extended at a larger number of wheat varieties and environmental conditions offering a more comprehensive influence of agro-climatic factors on the polymers characteristics. A focus on the variations of the polymer characteristics, excluding their influence on dough properties, is here proposed since many questions related to gluten health concern may be addressed to glutenin polymers. It is well-known that gluten may elicit adverse reactions in susceptible patients having celiac disease (CD) and wheat allergy (WA). Other people may suffer from gluten in absence of diagnosed CD and WA; this reaction was called non-celiac gluten sensitivity (NCGS) [26]. So far, no specific epitope of GSPs has been found as characteristic of NCGS. Other wheat grain components like α-Amylase and Trypsin Inhibitors (ATIs) Fermentable Oligo, Di-, Monosaccharides and Polyols (FODMAPS) are actively searched as triggering the so-called non-celiac wheat sensitivity (NCWS) (for review, see [27]). When gluten is analyzed, beside the quantitation and molecular characterization of the individual components (gliadins and glutenin) and quantitation of total polymers (%UPP), no physical characterization of the glutenin polymers is performed. The present study reports on some factors associated to the variation of the physical characteristics that could be useful for studying the gluten-related disorders. 

The main A4F-MALLS’ monomeric and polymeric protein profiles are specifically investigated here for the three major glutenin polymer characteristics: average molecular mass (Mw), gyration radius (Rw), and polydispersity index (PI). 

The aim of the present publication is to estimate the effect of allelic diversity of HMW-GS and LMW-GS and of environmental factors, like temperature and rainfall in particular, during GSP accumulation on specifically the polymer characteristics using a large set of cultivars grown in experimental fields. Climatic records over four years for 11 experimental locations in France, where 192 cultivars have been grown, provided the data, allowing for estimation of the genetic and environmental influences on the variation of the three major polymer characteristics. Consequences of the findings, including some current questions raised regarding non-celiac gluten sensitivity (NCGS), will also be discussed.

## 2. Materials and Methods 

### 2.1. Multilocal Trials

In the frame of collaborative research between INRA (Institut National de la Recherche Agronomique) and the majority of European private breeding companies operating in France, multi-local trials were carried out over four years: 2004–2006 (Program “Indice of Quality”) and 2008–2010 (Program “Tenacity–Extensibility Bread Loaf Volume”). The institutes Unilasalle and Arvalis were also associated with this research, led by INRA and financed by the FSOV (Fond de Soutien à l’Obtention Végétale). Many biochemical, genetic, and technological parameters were measured on the collected samples: 780 samples (130 cultivars in six locations) and 240 samples (68 different cultivars in total with 40 cultivars in six locations), respectively, in these two programs. Main results and presentations given can be consulted (https://www.fsov.org). Of the 12 locations, the Clermont Ferrand location was used three times, in 2004 and 2005, for “Indice of Quality” and in 2009 for “Tenacity–Extensibility Bread Loaf Volume.” To avoid overinfluence of the central area of France, one trial of the Clermont Ferrand location was removed. The trial harvested in 2005 was withdrawn since unusual climatic conditions (very high temperature and severe drought) occurred in that location for the three last months of wheat crop, causing shrivelled grains for the majority of cultivars. For the present study, a total of 885 samples harvested on 11 locations were used. The 11 locations are given in supplementary Table S1; two were identical (Clermont Ferrand in 2004 and 2009), and the nine others were different. Among the 198 cultivars (130 + 68), six cultivars that are not regularly replicated, were removed in our study. The 192 cultivars, originating mostly from France but also from some other European countries (UK, Netherland, and Germany), were hexaploidy winter bread wheat cultivars (registered varieties or homozygous stable cultivars) proposed by the INRA and breeding companies. They were not pedigree-related (sister lines). Experimental trials were located in the major areas of wheat production in France, spread between the North, West, and Central parts of France. Each trial was conducted in field experiments, including randomized plots (10 m^2^) with two replicates and using conventional agronomic practices, with mineral fertilization and fungicide protection, to achieve high grain yields without any water irrigation. The average nitrogen supply was around 155 Kg ha^−1^ and varied according the residual soil nitrogen available in the trials locations to ensure optimum grain production. The daily climatic records over the three last months of the wheat cycle (May, June, and July) provided by the closest climatic station of the experimental fields and have been registered. The sum of the temperatures and water precipitations averaged for May, June, and July each year are given in Supplemental Figures S1 and S2.

### 2.2. Technological Parameters

For each cultivar, the harvested grains of the two plots after cleaning were blended (50/50 *w*/*w*), and then, the resulting sample (approx. 1000 g) was maintained for at least three weeks at 15 °C and 65% relative hygrometry for moisture homogenization between samples. Moisture and test weight (TW) of the grain were measured in 400 g of sample using a Multigrain TR-Dj apparatus (Auburn, IL, 62615, USA). The thousand kernel weight (TKW) was determined according to the protocol AFNOR NF V03-702, now available under AFNOR NF EN ISO 520 [28]. Fifty grams of grain were whole-mealed using Cyclotec^TM^1093 (FOSS, Hillerød, Denmark) with a 0.75-mm sieve. The whole meal was used for PC and grain hardness (GH) determination by near-infrared spectroscopy (NIRS), as described by the AACC methods 39-11 and 39-70, respectively [29], using the Inframatic 8620 (Perten instruments, 67500, Haguenau, France). For each wheat sample, approximately 20 g of whole meal was placed in a closed vial at −20 °C for polymer extractions and analyses.

### 2.3. Separation of Glutenin Subunits by SDS-PAGE

The allelic diversity of HMW-GS and LMW-GS encoded at the three Glu-1 and Glu-3 loci was identified for the 192 cultivars experimented on over the four years. Proteins were extracted from five to ten individual seeds for each cultivar and submitted to SDS-polyacrylamide gel electrophoresis, as described by Branlard et al. [30]. The alleles, identified according to the nomenclature previously used, are given in Supplemental Table S1.

### 2.4. Proteomics 

Protein disulfide isomerase (PDI) data were collected from proteomics studies carried out by Tasleem-Tahir et al. [31] on developing endosperm using one cultivar “Récital” grown in a greenhouse in controlled conditions.

### 2.5. Asymmetric Flow Field-Flow Fractionation (A4F) Multi-Angle Laser Light Scattering (MALLS)

The principle of the separation technique and the wheat protein extraction protocol were previously described [20,22]. The polymer samples were analyzed with A4F-MALLS-UV using an Eclipse 3 A4F Separation System controller (Wyatt Technology, Toulouse, France) serially connected to a UV detector and a Wyatt DAWN HELEOS 18-angle MALLS detector (Wyatt Technology, Toulouse, France). The MALLS detector operated at a wavelength of 690 nm. An Agilent 1100 series isocratic pump (Agilent Technologies, Les Ulis, France) with an in-line vacuum degasser and an Agilent 1100 series autosampler delivered the eluent flow and handled the sample injection (30 µL) into the A4F separation channel. The UV detector was set at 214 nm. At the pump outlet, a regenerated cellulose filter with a pore size of 0.2 µm (Merck Millipore, Guyancourt, France) was installed, ensuring that particle-free carrier entered the channel. The A4F channel had a trapezoidal geometry (tip-to-tip length of 28.6 cm, and inlet and outlet widths of 2.15 and 0.6 cm, respectively). The spacer nominal thickness was 350 μm. The ultrafiltration membrane, forming the accumulation wall, was a regenerated cellulose membrane with a nominal cut-off of 10 kDa (Merck Millipore, Guyancourt, France). Validation of the A4F system performance and normalization of MALLS detector was performed with monomeric bovine serum albumin solubilized in the mobile phase (Sigma-Aldrich, Saint-Quentin Fallavier, France). The carrier liquid was sodium phosphate buffer (0.05 M, pH 6.9) containing 0.1% (*w*/*v*) SDS and was filtered through a 0.1-µm membrane (Merck Millipore, Guyancourt, France). The fractionation method used a constant detector flow of 1 mL/min and a gradient cross-flow. The method started with a focus time of 0.5 min at a flow rate of 2 mL/min. This step was followed by a focus/injection time of 1.0 min at 0.2 mL/min and a relaxation/focusing time of 0.5 min. Elution then followed at an outflow rate of 1.0 mL/min and with a cross-flow rate decreasing exponentially from 3.0 to 0.0 mL/min for 14 min. Finally, elution at a cross-flow rate of 0.0 mL/min was maintained for 9 min. The recorded MALLS and UV data were processed using Astra software (v. 5.3.4.13, Wyatt Technology, Santa Barbara, CA, USA). The Mw and radius were obtained by fitting the MALLS data using the Berry method, performing a second order fit to the data collected at scattering detectors 5–17.

Molar mass and radius can be obtained by MALLS combined with concentration measurements by applying the Rayleigh-Gans-Debye approximation [32] (Equation (1)):

(1) $$\left(\frac{K^{*}c_{i}}{R_{\theta }}=\frac{1}{M_{i}P\left(\theta \right)}+2A_{2}c\right)$$

where $R_{\theta }$ is the excess Rayleigh scattering over and above that of the solvent due to a solute of concentration c_i_ for slice i of the fractionated sample. $K^{*}$ is an instrumental constant dependent on the wavelength and refractive index increment (dn/dc) of the solute in the particular solvent. *M_i_* is the weight-average molar mass for the solute. P(θ) is the scattering function that describes the angular dependence of the scattered light intensity from which the mean square radius <*r2*> is derived. $A_{2}$ is the second virial coefficient and captures nonideality due to solvent-solute interactions; it is expected to be vanishingly small in a dilute fractionated sample.

(2) $$\left(Mw=\frac{\Sigma c_{i}M_{i}}{\Sigma c_{i}}\right)$$

(3) (Mn=ΣciΣciMi)

(4) $$\left(Mz=\frac{\Sigma c_{i}M_{i}{}^{2}}{\Sigma c_{i}M_{i}}\right)$$

(5) $$\left(Rw=\frac{\Sigma \left(c_{i}{\langle r2\rangle }_{i}\right)}{\Sigma c_{i}}\right)$$

(6) $$\left(Rn=\frac{\Sigma \left(\frac{c_{i}}{M_{i}}{\langle r2\rangle }_{i}\right)}{\Sigma \left(\frac{c_{i}}{M_{i}}\right)}\right)$$

(7) $$\left(Rz=\frac{\Sigma \left(c_{i}M_{i}{\langle r2\rangle }_{i}\right)}{\Sigma \left(c_{i}M_{i}\right)}\right)$$

The average weight-average molar mass (Equation (2)) is derived by measuring $R_{\theta }$ at multiple angles for each slice *i* of the fractionated sample. The concentration of each slice, $c_{i}$, is measured by UV detector. The number-average molar mass is defined as Equation (3). The z-average molar mass is defined as Equation (4). The *PI* is calculated as follows: *PI = Mw*/*Mn*. The weight-average mean square radius is calculated as Equation (5). The number-average mean square radius is determined as Equation (6). Lastly, the z-average root mean square radius *Rz* is derived from the mean square radii (Equation (7)).

### 2.6. Statistics 

All statistical analyses (i.e., descriptive statistics, simple regression, Pearson correlation, ANOVA with general linear model (GLM), and partial least square regression (PLS)) were performed using Statgraphics^®^ software. The broad-sense heritability, *H^2^*, values of the technological parameters and polymer characteristics were computed using the GLM of variance analysis, considering cultivars grown on several locations in a given year as the random variate. The heritability was computed as described in Equation (8), where $\sigma^{2}g\;$ is the estimated genetic variance, and $\sigma^{2}r$ the residual variance. The *H^2^* values reported in Table 1 were averaged over the four experimental years.

(8) H2=σ2g(σ2g+σ2r)

## 3. Results and Discussion

### 3.1. Large Phenotypic Variation of Quality Parameters

The phenotypic values measured on the 885 samples (i.e., 130 × 5 + 40 × 6 − 5 lacking samples) exhibited a wide range of variation for all traits measured. The grain PC was between 8.7% and 15.1% of dry matter (dm) and the GH was from very soft to very hard types of wheat. The range of variation was from 29.0 g to 64.9 g for TKW and from 64.0 kg hL^−1^ to 86.3 kg hL^−1^ for TW, evidencing that genetic and growing environmental factors had impacted the grain filling (Table 1). The highest phenotypic variations were found for the characteristics of the polymers: 1.1 to 48.0 MDa for Mw, 6.7 to 116.2 nm for Rw, and 1.04 to 82.94 for PI. The polymer characteristics were not basically associated to PC, since Pearson correlations, computed per location, revealed that, out of the 11 locations, only one, five, and two locations had significant correlations (*p* < 0.05) with Mw, Rw, and PI, respectively. The three characteristics were not associated to GH, TKW, and TW as well.

### 3.2. The Polymer Characteristics Responded More to Environmental Factors than Genetic Factors

ANOVA, when computed with genotypes, locations, or years, revealed the maximum variance explained by each of these factors used as unique explanatory variate. As expected, genetic differences were prominent in GH variations, but the percentage for TW, although significant, was low. Of particular interest for the polymer characteristics, the part of phenotypic variance explained by genotypes are relatively high: 55.0%, 79.6%, and 61.5% for Mw, Rw, and PI, respectively. The phenotypic variance of each polymer characteristic was better explained by environmental factors; greater *R*^2^ percentages were found for the locations or for the years of experimental trial (Table 1). This higher influence of environmental factors, as compared to the genetic factor, initially noticed for polymeric proteins [20] is also confirmed here by the broad-sense heritability calculus. The average H^2^ coefficients computed for the polymer characteristic were similar or lower than the H^2^ of PC, well-known to be largely influenced by environment. Although the years and locations were of higher effectiveness than cultivars on the phenotypic variations, the multiple comparisons performed between the 192 cultivars showed that at least three distinct groups of cultivars (of low, medium, or high value) could be obtained for each polymer characteristic (data not shown). Being of low heritability, the polymer characteristics can be employed, however, for phenotypic evaluation and cultivar ranking in the breeding program. The analysis of the alleles at the six glutenin loci, encoding HMW-GS and LMW-GS, offered the opportunity to better approach their genetic influence on the phenotypic variation of polymer characteristics.

### 3.3. The Allelic Diversity of Glutenins had Limited Influence on Polymer Characteristics

The PLS regression first evidenced that PC and GH had very low, but significant, influences on Mw and PI characteristics (Table 2). Although all experimental plots received optimum nitrogen supply at each location for high grain yield, other agro-climatic conditions may have induced differences in nitrogen absorption and remobilization in spikes. Higher temperatures and droughts are known to reduce nitrogen absorption by the plant and starch content in grains and, also, to shorten the duration of the grain-filling period [33]. The increased PC is, consequently, opposed to higher protein accumulation and polymerization, requiring longer normal accumulation time. Once PC and GH are involved, the PLS regression revealed that LMW-GS diversity contributed two times more to polymer characteristics than HMW-GS. These global contributions of the HMW-GS and LMW-GS to polymer characteristics are in full agreement with the quantitative proportion classically found of the two types of glutenins: i.e., 1/3 and 2/3, respectively. The 31 alleles encoding the HMW-GS and LMW-GS increased the explanation of the phenotypic values by only 17.1%, 25.4%, and 16.8% for Mw, Rw, and PI, respectively. Similar contributions (not shown in [24]) were found for the 28 alleles present in the 68 cultivars experimented in 2008–2010. Among the 14 alleles encoding HMW-GS, the Glu-A1 null allele, all the Glu-B1 alleles, and the subunits Glu-D1 2–12 had negative influences on polymer characteristics. In contrast, the subunits Glu-A1 1, Glu-D1 3–12, and Glu-D1 5–10 increased the three characteristics. For the 17 alleles encoding LMW-GS, Glu-A3 a and b had negative influences, whereas Glu-A3 e or f, noted “ef”, were positively associated. For the Glu-B3 locus, alleles a, b, and i were negatively associated, whereas g and j were positively associated to the three characteristics. The alleles GluD3 a and c (Glu-D3 b) were negatively (positively) associated to the three characteristics.

The standardized coefficient, described by the PLS regression, for the 14 HMW-GS alleles are shown in Supplemental Figure S3a–c and for the 17 LMW-GS alleles in Supplemental Figure S3d–f. Altogether, PC, GH, and glutenin diversity explained only 20%, 25%, and 17% of the phenotypic diversity of Mw, Rw, and PI, respectively. When rainfalls registered in each experimental location for May, June, and July were introduced into the PLS regression, the total part of the variance explained reached 28.3%, 39.4%, and 37.1%, respectively (Table 2). When the cumulative daily average temperatures over the months (Tmeans of May, June, and July) computed in the 11 locations were added to glutenin alleles as explanatory variates, the percentages were two times those obtained with the rainfall averages. The total phenotypic variations explained were 60.5%, 73.6%, and 76.4%, for Mw, Rw, and PI, respectively (Table 2).

**Figure 1:**
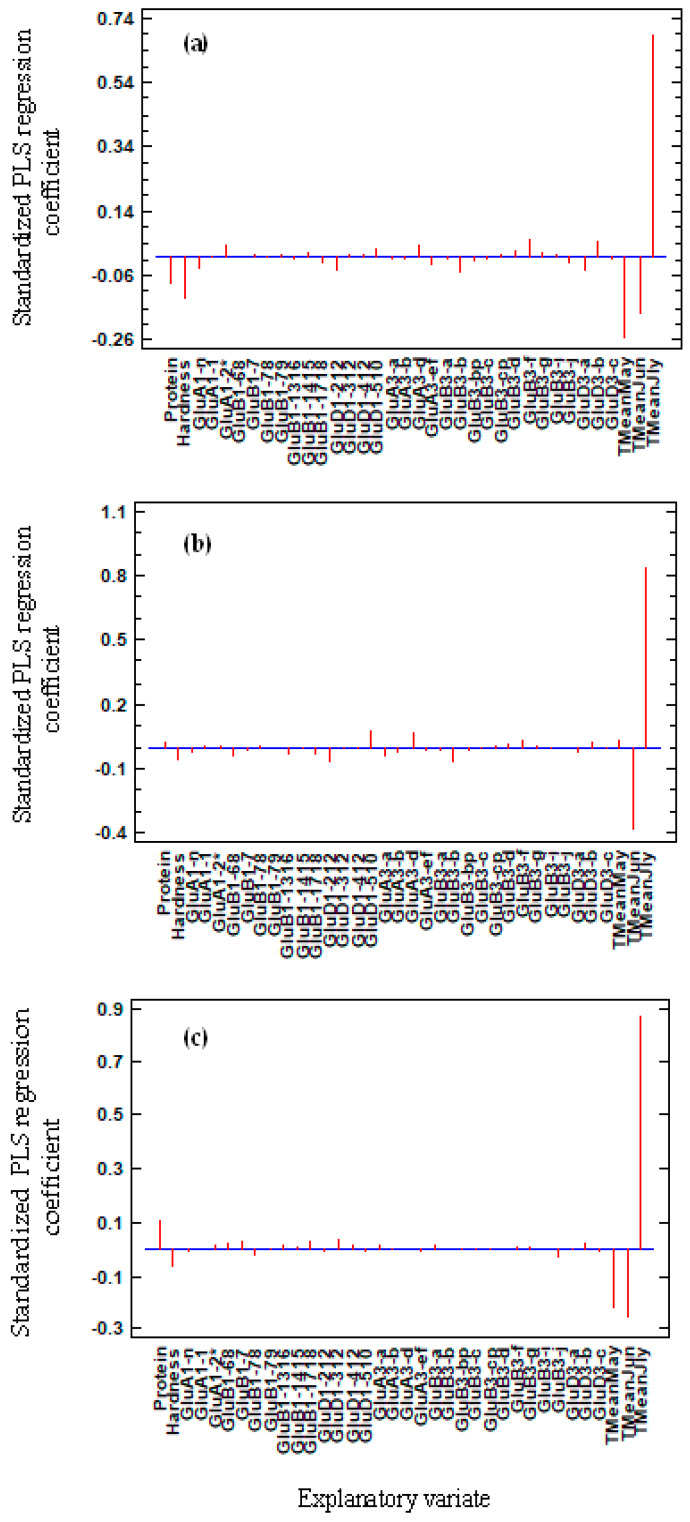
Standardized coefficients of the partial least square (PLS) regressions, explaining the molecular mass (Mw) (**a**), gyration radius (Rw) (**b**), and polydispersity index (PI) = Mw/Mn (**c**) characteristics of the polymers. The grain protein concentration (PC) (noted Protein) and grain hardness (GH) (noted Hardness) were explanatory variates first introduced in regression, followed by 14 alleles of high molecular weight glutenin subunits (HMW-GS); 17 alleles of low molecular weight glutenin subunits (LMW-GS); and the average temperatures of May, June, and July (noted Tmean May, Tmean June, and Tmean July).

The standardized coefficient attributed to each of the explanatory variates clearly evidenced the low impact of the different glutenin alleles compared to the monthly averaged temperature of the last three months of wheat crop in the 11 locations for Mw, Rw, and PI (Figure 1a–c, respectively). Then, among the three variates (Tmean May, Tmean Jun, and Tmean July,) the latest had, by far, the largest influence on increasing the polymer characteristics.

### 3.4. The Temperature during Grain Maturation Had a Major Influence on Polymers’ Characteristics

Without taking into account the influence of the PC, GH, and glutenin alleles on polymer characteristics, the cumulative daily average temperatures over the month of July were used as unique explanatory variates to explain the polymer characteristics. The best regression, computed to maximize the R^2^, was of a linear type for Rw and of an exponential type for Mw and PI. 

**Figure 2:**
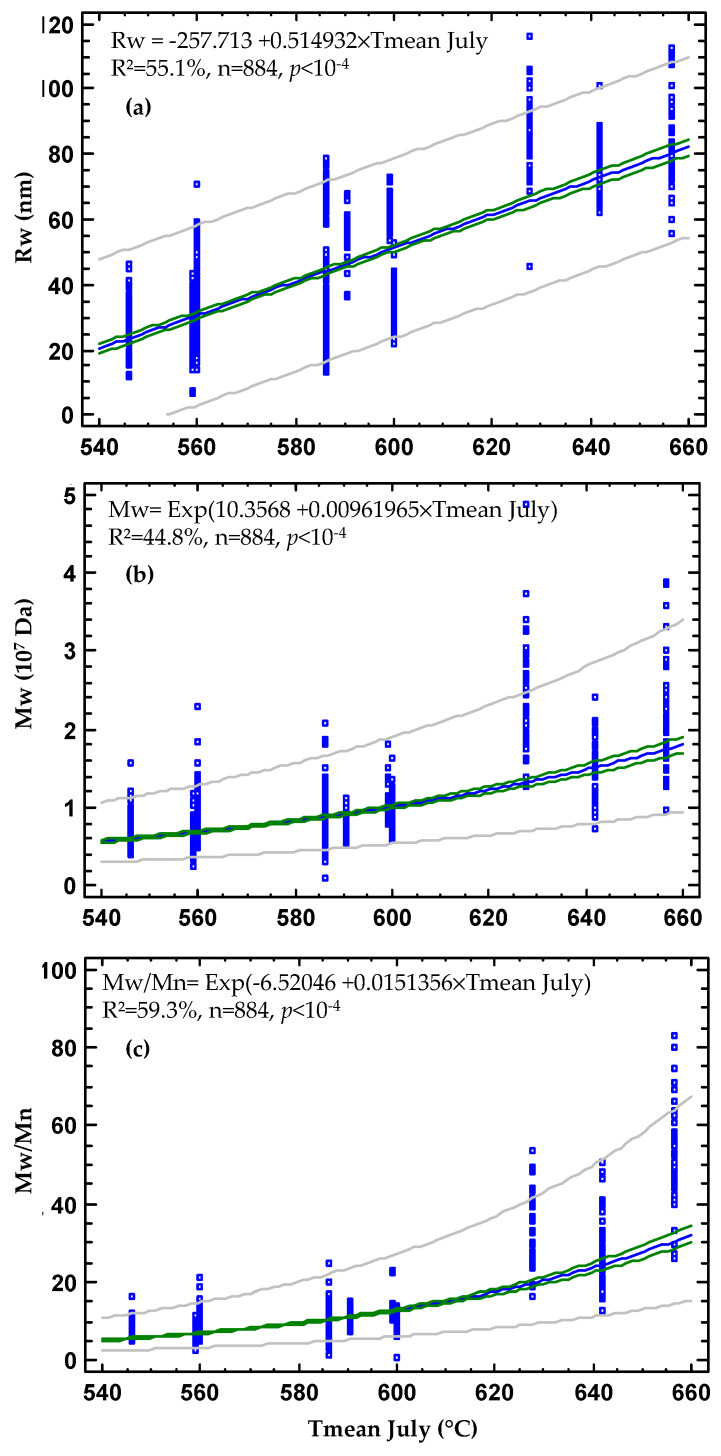
Regressions between the characteristics of the glutenin polymers, measured using Asymmetric Flow Field-Flow Fractionation Multi-Angle Laser Light Scattering (A4F-MALLS), and the cumulative daily mean temperatures (°C) of July (noted Tmean July) in the 11 wheat trial locations. (**a**) Rw of polymers (nm), (**b**) Mw of polymers (Da), and (**c**) PI of polymers (PI = Mw/Mn). Two locations with similar Tmean values (July, Verneuil l’Etang 2005: 586 °C and Mons en Pévèle 2009: 586.1 °C) are not separated on the figures. R^2^: percentage of phenotypic variance.

As shown in Figure 2, the cumulative daily average temperatures over the month of July explained 55.1%, 44.8%, and 59.3% (for each, *n* = 885 and *p* < 10^−4^) of the phenotypic variations of Rw, Mw, and PI, respectively. These regressions clearly evidence the influence of temperature in the experimental field trial during the last month of the wheat crop, which is highly associated to increasing the polymer characteristics. Other regressions performed using either the average temperature of May or of June or the average temperatures cumulated during the 50 days usually acquired from grain formation to maturity did not explain as much of the polymer characteristics as did the average temperature of the month of July. The vertical dots, corresponding to phenotypic values measured in each location, on Figure 2 result partly from genetic differences between cultivars, which differed for the PC, GH, and glutenin compositions. Nevertheless, as reported in Table 2, a significant part of about 40%, 27%, and 23% of the phenotypic variations of Mw, Rw, and PI, respectively, remain to be explained. The cultivars did not have similar earing precocities, possibly causing different adapted responses to higher temperatures during grain accumulation. In addition, although the A4F-MALLS measurements were replicated, due to a lack of measures on field replicates for all cultivars, the interactions (cultivars × locations) were not tested for the 192 cultivars on the 11 locations. Several significant interactions were reported between GH (PinB alleles) and HMW-GS (Glu-A1 alleles or Glu-A3 alleles) for the three polymer characteristics with cultivars grown in 2008–2010 [24]. From a practical approach, when the PC and GH of the grain sample, measured by NIRS, are introduced into the regression, together with the average temperature of the last month of the wheat crop (i.e., July), the above percentages were almost unchanged: 58.4%, 45.0%, and 60.4% for Rw, Mw, and PI, respectively. 

The highly significant increasing polymer characteristics, associated with different average temperatures from the month of July, were computed from hourly (night and day) daily temperatures recorded in locations where the multilocal trials were implanted: three locations in 2004, two in 2005, three in 2009, and three in 2010. The difference between lowest sum of the daily average temperatures in July (546 °C) registered at La Chapelle d’Armentière (North of France) in 2004, and the highest average temperature (656.6 °C) registered at Clermont Ferrand (Central France) in 2010 was 110.6 °C. It corresponds to a difference of only 3.5 °C of the daily average temperature between the two experimental sites (17.6 °C versus 21.1 °C). When introduced in the fitted equations (Figure 2), this 3.5 °C difference caused tremendous changes in the characteristics of the polymers. It increased the average Rw of the polymers (from 23.4 nm to 80.4 nm) by 242%, the average Mw of the polymers (from 6.0 × 10^6^ Da to 17.4 × 10^6^ Da) by 189%, and the PI of the polymers (from 5.71 to 30.49) by 434%.

Some approaches may provide possible explanations of the observed phenomenon. Many technological studies have reported the influence of experimentally monitored temperatures on wheat flour proteins—for example on albumins and globulins on gluten and on flour protein during extrusion cooking (see [34] for review). All these studies have shown the strong impact of thermal energy input on the formation of SS crosslinks. However, the temperature often used (70 °C or more) exceeded the highest peaks recorded in our multilocal trials. The two highest temperatures were recorded in the North of France for only one day in July: 32.4 °C at Estrée Saint Denis in 2009 and 35.4 °C at Cappelle en Pévèle in 2010. In addition, the characteristics of the polymers, such as Rw, Mw, and PI, could not be measured with HPLC methods. 

### 3.5. Proteomics Studies Indicate a Possible Stress during Protein Accumulation

**Figure 3:**
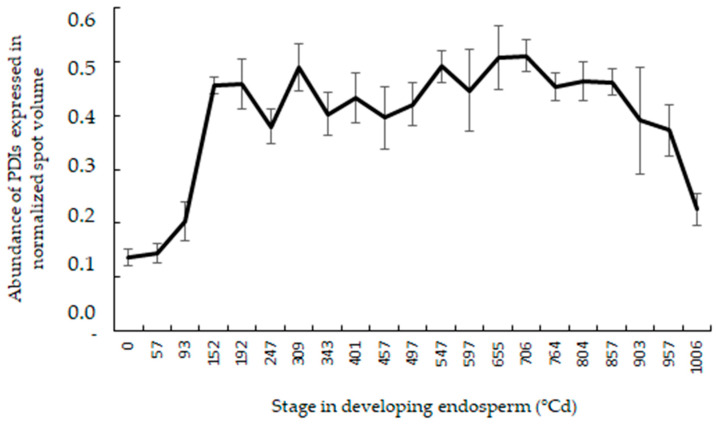
Evolution of the average abundance of eight protein disulfide isomerases (PDIs) (± SD) expressed in the endoplasmic reticulum (ER) at 21 stages in developing endosperm (from 0 to 1006 °Cd) of cultivar ”Récital” grown in controlled-temperature environments (computed from [31]).

The strong influence of temperature on the polymer characteristics may result from higher activities of the oxidative molecules catalyzing the formation of SS. Among these molecules in the wheat endosperm, we may find the PDI, sulfhydryl oxidases, and reactive oxygen species (ROS), like hydrogen peroxide, dehydroascorbate, and oxidized glutathione. Proteomics analyses of developing wheat endosperm at 21 stages led to the identification of eight PDIs, specifically present in the endoplasmic reticulum, where polymerization occurs: one PDI-2 precursor, three PDIs, and four PDI-likes [31]. Expression of abundance of each of these eight PDIs over the 21 stages (between 0 °Cd and 1006 °Cd) were recorded. The curve of the average expression of these eight PDIs clearly shows a decline of the PDIs’ abundance after 700 °Cd (Figure 3).

During the 15 last days of grain maturation, the average abundance of PDIs dropped to 55.8%. This decline was recorded for unstressed wheat grown in a controlled environment where air temperature close to the ears did not exceed 24.2 °C [31], far below the peak temperatures registered over the four years of the experiment.

Protein disulfide isomerase (PDI) is a multifunctional redox chaperone of the endoplasmic reticulum (ER) [35]. PDI is a protein folding catalyst and is also responsible for the isomerization, formation, and rearrangement of protein disulfide bonds, thereby providing another mechanism by which native protein conformation is maintained. We may hypothesize that, when temperature increases, the free thiols of glutenins are oxidized until the amount of ROS is not critical. Correct folding and assembly of nascent proteins in ER, aided by chaperones and cochaperones like binding protein (BiP) and PDI, prevents the aggregation of proteins [36]. Accumulation of storage proteins in endosperm creates a heavy load in the secretory pathway and unfavorable environmental conditions, such as heat stress, drought, or pathogen attack, can upset the ER quality control system. When the imbalance between protein load and folding machinery occurs, a stress signal triggers a cellular response, the unfolded protein response (UPR).

In wheat stress-related proteins, characteristics of the UPR were found in both soft and hard near-isogenic lines, these stress-related proteins being more abundant in the hard ones [37]. Although no proteomics–transcriptomics studies were carried out over the course of our experiment, the current knowledge strongly supports the idea that higher abnormal polymers result from UPR occurring in wheat endosperm. Higher polymer Mw (up to 234 × 10^6^ Da)—that is, more than 50 times the classic Mw, were recently measured on wheat grown in France where both the high temperature and drought were prevalent [38]. Molecular mechanisms associated with abnormal polymerizations, that are here revealed as resulting from high temperatures, need to be fully analyzed using both transcriptomics and proteomics tools on wheat grown in fully controlled environments. These investigations are obviously needed not only for technological purposes but, also, for human health.

When wheat varieties are studied for human health, the amount of insoluble polymers is usually measured by SE-HPLC. This amount, often closely related to the grain PC, gives no measure of polymer Mw. These glutenin polymers are hydrophobic and heat-resistant. Their size is often further increased by oxidation resulting from intense kneading, and they are only partially hydrolyzed during the short fermentations generally practiced today for many baked products. Are these gluten proteins easily hydrolyzed if they are packed in a huge polymer mass?

Numerous approaches were developed to reduce the gluten exposure for celiac and sensitive persons [34,39], like: screening in the wheat gene pool and pyramiding null alleles, the downregulation of specific GSPs, and the insertion of exogenous proteinases sequences in wheat genomes for specific peptides hydrolysis or better gluten digestion. Some adaptations for technological processes were also proposed, like milling, use of a microwave, sourdough fermentation, etc. The final goal being the true digestibility of the gluten. Today, nutritionists may use a dedicated tool to measure the polymer mass that could be easily digestible for sensitive persons.

## 4. Conclusions

Global warming is observed on all continents where wheat is produced, from varieties adapted to each country and originating from different pedigrees. The high temperatures registered in France during our four experimental years are encountered in many other countries, as well. They cause an increase in the molecular weights of glutenin polymers, which appear to be a likely hypothesis behind non-celiac gluten sensitivity (NCGS). No glutenin epitope specific to this sensitivity has been revealed to date. Consequently, several alternative hypotheses are currently being investigated to explain NCGSs [26,27]. However, we notice that polymers characteristics of glutenins (Mw, Rw, and PI) have not been investigated, thus far, by wheat geneticists, nor by technologists, food manufacturers, or nutritionists. In addition, up until now, none of the studies devoted to NCGS [27] have measured the polymer Mw and the impact of their variations on human health. The tremendous increase of glutenin polymer dimensions, revealed here, in response to high temperatures strongly encourages all scientists involved in wheat consumer health studies to use suitable tools, like A4F-MALLS, to better characterize the glutenins’ polymers. Obviously, many studies on the genetic and molecular mechanisms occurring in ER and the proteins’ bodies remain to be undertaken, ultimately having some influence on those characteristics (Mw, Rw, and PI). For the moment, the focus on dough fermentation seems to be the quickest way.

## Figures and Tables

**Table 1:** Values of statistical distribution of the 192 cultivars experimented in 11 locations for protein content (PC); grain hardness (GH); thousand kernel weight (TKW); test weight (TW); and the polymer characteristics: molecular mass (Mw), gyration radius (Rw), and polydispersity index (PI). The percentage (*R*^2^%) of phenotypic variance was obtained using one factor ANOVA, and the heritability H^2^ coefficient was averaged over the H^2^ computed per experimental year.

Parameters (Unit)	Total Samples	Mean ^1^	S.D. ^2^	Min ^3^	Max ^4^	R^2^ % Genotype (192)	R^2^ % Location (11)	R^2^ % Year (4)	H^2^ (4)
PC (%dm)	883	11.4	1.02	8.7	15.1	25.6	42.1	0	24.6
GH	883	51.9	23.66	−6.3	112.8	83.8	9.1	4.8	78.7
TKW (g)	883	45.9	4.52	29.0	64.9	45.9	26.4	7.3	52.3
TW (kg/hL)	883	78.5	3.57	64.0	86.3	4.3	59.6	56.3	35.0
Mw (kDa)	885	9554.4	5485.6	1142.0	48,777.5	55.0	70.1	59.1	11.8
Rw (nm)	885	42.4	20.94	6.7	116.2	79.6	85.2	82.6	25.0
PI (Mw/Mn)	885	12.58	11.81	1.04	82.94	61.5	85.6	74.1	21.2

(1) Mean value, (2) Standard Deviation, (3) Minimum value, (4) Maximum value.

**Table 2:** Partial least square (PLS) regression: Part of the phenotypic variance of molecular mass (Mw), gyration radius (Rw) and polydispersity index (PI) explained by grain protein concentration (PC), grain hardness (GH), high molecular weight glutenin subunits (HMW-GS) alleles, low molecular weight glutenin subunits (LMW-GS) alleles, cumulative water precipitations (WatSum), and cumulative mean temperatures (Tmean)for the three final months in the 11 experimental fields of wheat crops.

Explanatory Variate	Mw	Rw	PI = Mw/Mn
	(%)	(%)	(%)
PC + GH	3.628 *	0.064 NS	0.688 *
PC + GH	8.846 *	8.807 *	5.316 *
+HMW-GS			
PC + GH	16.465 *	17.630 *	14.419 *
+LMW-GS			
PC + GH	20.781 *	25.498 *	17.570 *
+HMW-GS			
+LMW-GS			
PC + GH	28.342 *	39.350 *	37.052 *
+HMW-GS			
+LMW-GS			
+ WatSum May			
+ WatSum Jun			
+ WatSum Jly			
PC + GH	60.485 *	73.627 *	76.441 *
+HMW-GS			
+LMW-GS			
+ Tmean May			
+ Tmean Jun			
+ Tmean Jly			
PC + GH	65.436 *	79.146 *	84.310 *
+HMW-GS			
+LMW-GS			
+ WatSum May			
+ WatSum Jun			
+ WatSum Jly			
+Tmean May			
+ Tmean Jun			
+ Tmean Jly			

(*) significant at *p* < 0.05; NS: not significant.

## Supplementary Materials

The following are available online at https://www.mdpi.com/2304-8158/9/5/683/s1: Figure S1: Mean, Min, and Max of the sum of the accumulated daily temperatures per month (°C) for May, June, and July in the 3 experimental locations in 2004, 2 locations in 2005, and 3 in 2009 and 2010, Figure S2: Mean, Min, and Max of the sum of the accumulated water precipitations per month for May, June, and July in the 3 experimental locations in 2004, 2 locations in 2005, and 3 in 2009 and 2010, Figure S3: Standardized coefficients of the PLS regressions aimed to explain the Mw, Rw, and PI = Mw/Mn characteristics of the polymers, Table S1: Locations and seed companies involved in multilocal trials, Table S2: Frequencies of alleles encoding HMW-GS an LMW-GS in the 192 wheat cultivars.
