# Supplementary material for: Genetic and Environmental Factors Associated to Glutenin Polymer Characteristics of Wheat
Source: Foods. 2020 May 25;9(5):683. doi: 10.3390/foods9050683 (PMC7278847; doi:10.3390/foods9050683)
Supplement: Supplementary file 1 [file foods-09-00683-s001.pdf]

Supplemental Tables and Figures

Table S1. Locations and seed companies involved in multilocal trials.

Years	Locations in France	Seed Company or Institute
**2003–2004**	La Chapelle d'Armentières, 59930	Serasem
	Orgerus, 78910	CC-Benoist- Syngenta
	Clermont Ferrand, 63100	INRA
**2004–2005**	Cappelle en Pévèle, 59242	Florimond-Desprez
	Verneuil l'Etang 77390	Verneuil Recherche -LVH
**2008–2009**	Estrées St Denis, 60190	Saaten Union
	Mons en Pévèle 59246	Momont-Hennette
	Orgerus, 78910	Syngenta Seeds
**2009–2010**	Cappelle en Pévèle, 59242	Florimond-Desprez
	Clermont Ferrand, 63100	INRA
	Reclainville, 28150	Caussade Semences

Table S2. Frequencies of alleles encoding HMW-GS an LMW-GS in the 192 wheat cultivars.

**Glu-A1**	GluA1-n	GluA1-1	GluA1-2*							
	**44.8**	**37.3**	**17.9**							
**Glu-B1**	GluB1-68	GluB1-7	GluB1-78	GluB1-79	GluB1-1316	GluB1-1415	GluB1-1718			
	**21.5**	**6.2**	**27.9**	**21.4**	**4.4**	**2.0**	**15.5**			
**Glu-D1**	GluD1-212	GluD1-312	GluD1-412	GluD1-510						
	**34.1**	**8.2**	**4.5**	**53.2**						
**Glu-A3**	GluA3-a	GluA3-b	GluA3-d	GluA3-ef						
	**36.7**	**0.6**	**33.8**	**28.9**						
**Glu-B3**	GluB3-a	GluB3-b	GluB3-bp	GluB3-c	GluB3-cp	GluB3-d	GluB3-f	GluB 3-g	GluB 3-i	GluB 3-j
	**2.3**	**11.0**	**5.0**	**11.5**	**3.8**	**1.8**	**8.9**	**53.6**	**0.6**	**1.6**
**Glu-D3**	GluD3-a	GluD3-b	GluD3-c							
	**3.4**	**8.7**	**88.0**							

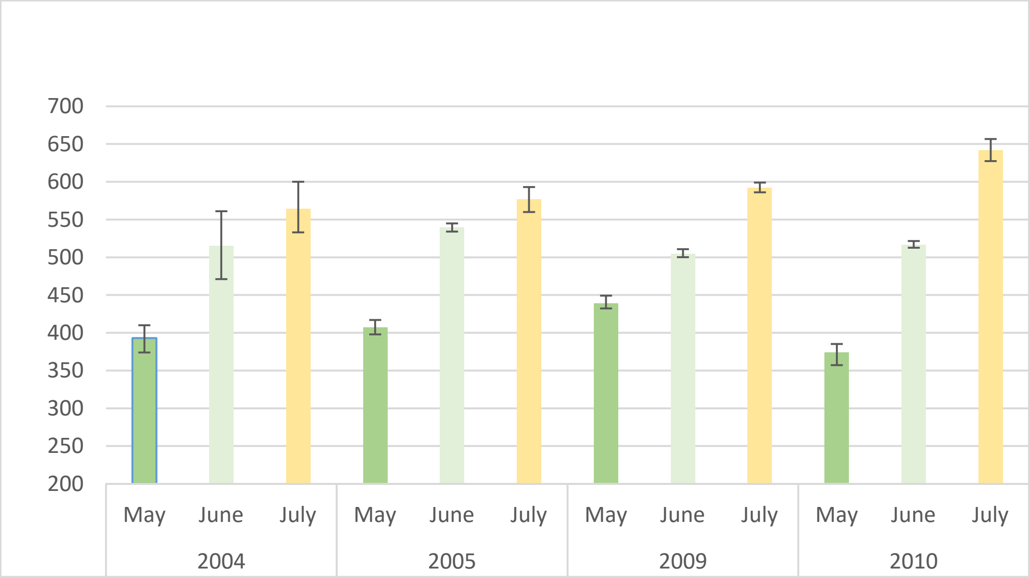

Figure S1. Mean, Min and Max of the sum of the cumulated daily temperature per month for May, June and July in the 3 experimental locations in 2004, 2 in 2005, 3 in 2009 and 3 in 2010.

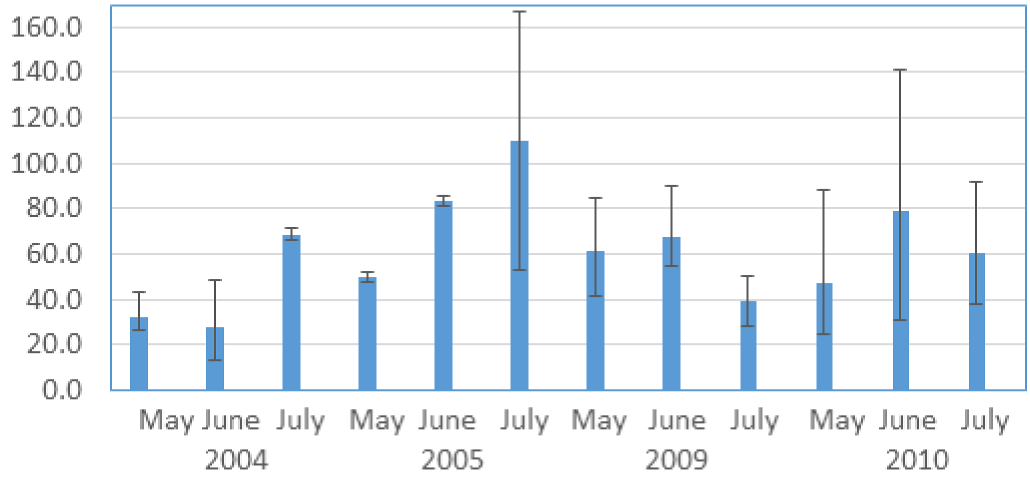

Figure S2. Mean, Min and Max of the sum of the cumulated water precipitations per month for May, June and July in the 3experimental locations in 2004, 2 in 2005, 3 in 2009 and 3 in 2010.

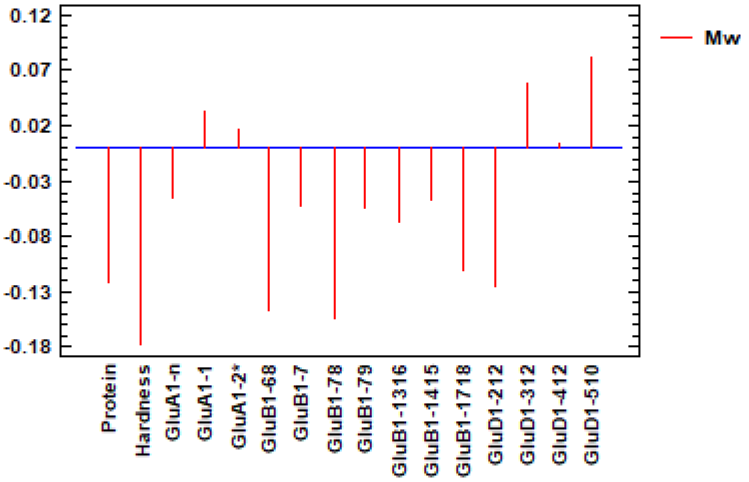

Mw
0.12
0.07
0.02
-0.03
-0.08
-0.13
-0.18
Protein
Hardness
GluA1-n
GluA1-1
GluA1-2*
GluB1-68
GluB1-7
GluB1-78
GluB1-79
GluB1-1316
GluB1-1415
GluB1-1718
GluD1-212
GluD1-312
GluD1-412
GluD1-510

3a

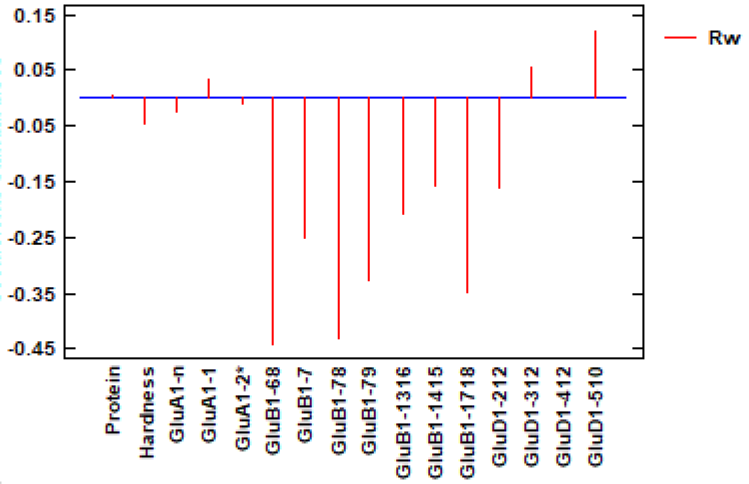

Rw
0.15
0.05
-0.05
-0.15
-0.25
-0.35
-0.45
Protein
Hardness
GluA1-n
GluA1-1
GluA1-2*
GluB1-68
GluB1-7
GluB1-78
GluB1-79
GluB1-1316
GluB1-1415
GluB1-1718
GluD1-212
GluD1-312
GluD1-412
GluD1-510

3b

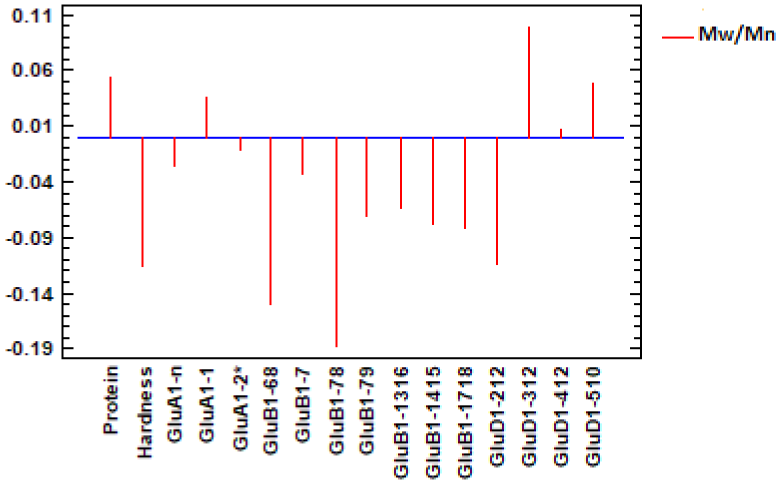

Mw/Mn
0.11
0.06
0.01
-0.04
-0.09
-0.14
-0.19
Protein
Hardness
GluA1-n
GluA1-1
GluA1-2*
GluB1-68
GluB1-7
GluB1-78
GluB1-79
GluB1-1316
GluB1-1415
GluB1-1718
GluD1-212
GluD1-312
GluD1-412
GluD1-510

3c

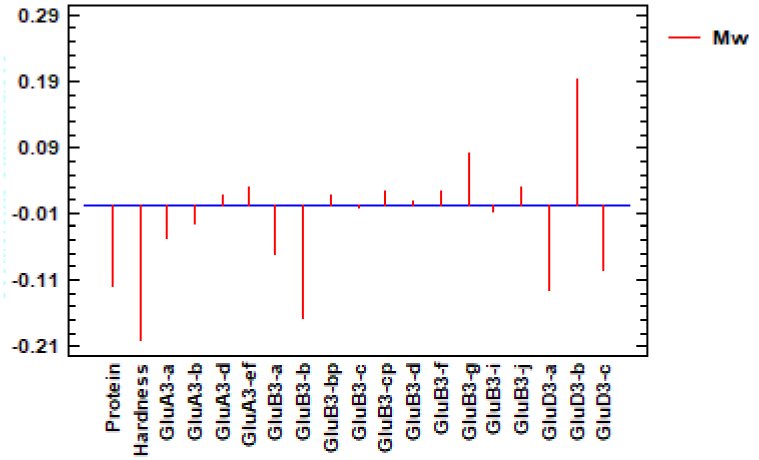

3d

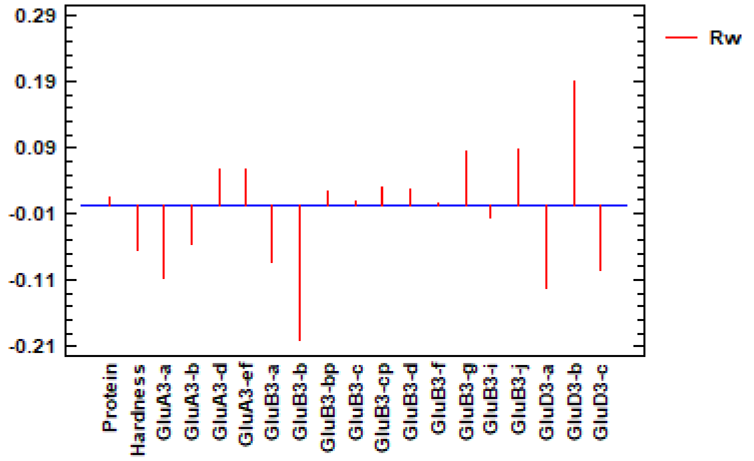

3e

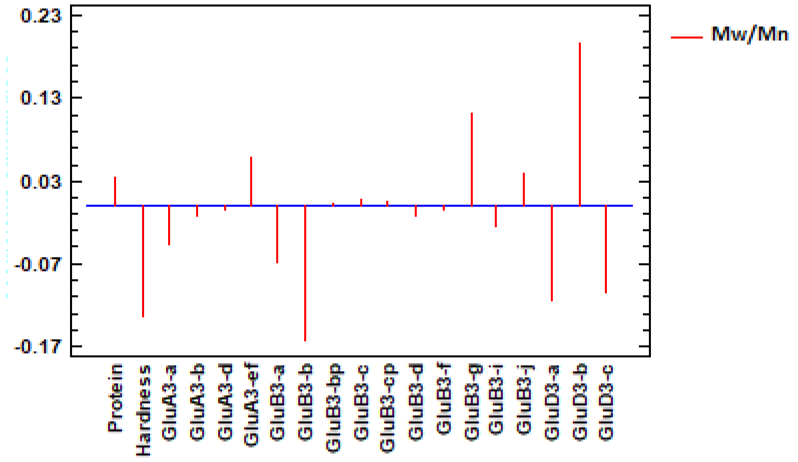

3f

Figure S3. Standardized coefficients of the PLS regressions aimed to explain the MW, Rw and PI= Mw/Mn characteristics of the polymers. The protein concentration of the grain PC (noted protein) and grain hardness GH (noted hardness) were explanatory variates first introduced in regression then alleles of HMW-GS (**a**–**c**, respectively) or alleles of LMW-GS (**d**–**f**, respectively).
